# Supplementary material for: Situation Analysis of Early Implementation of Programmatic Management of Tuberculosis Preventive Treatment among Household Contacts of Pulmonary TB Patients in Delhi, India
Source: Trop Med Infect Dis. 2024 Jan 17;9(1):24. doi: 10.3390/tropicalmed9010024 (PMC10818279; doi:10.3390/tropicalmed9010024)
Supplement: Supplementary file 1 [file tropicalmed-09-00024-s001.zip › tropicalmed-2707987-supplementary.pdf]

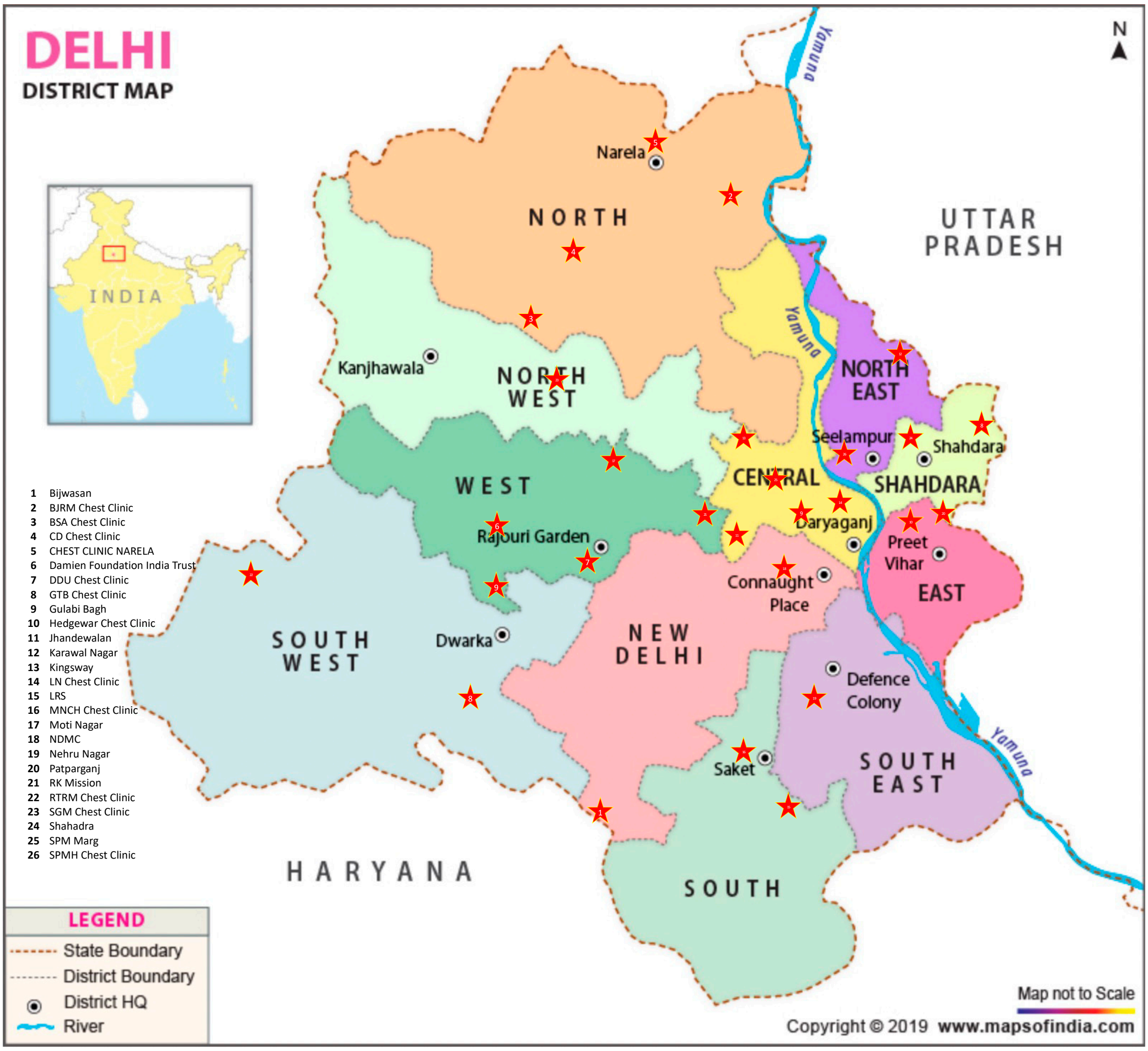

Figure S1. Map of Delhi State along with 25 Chest clinics.

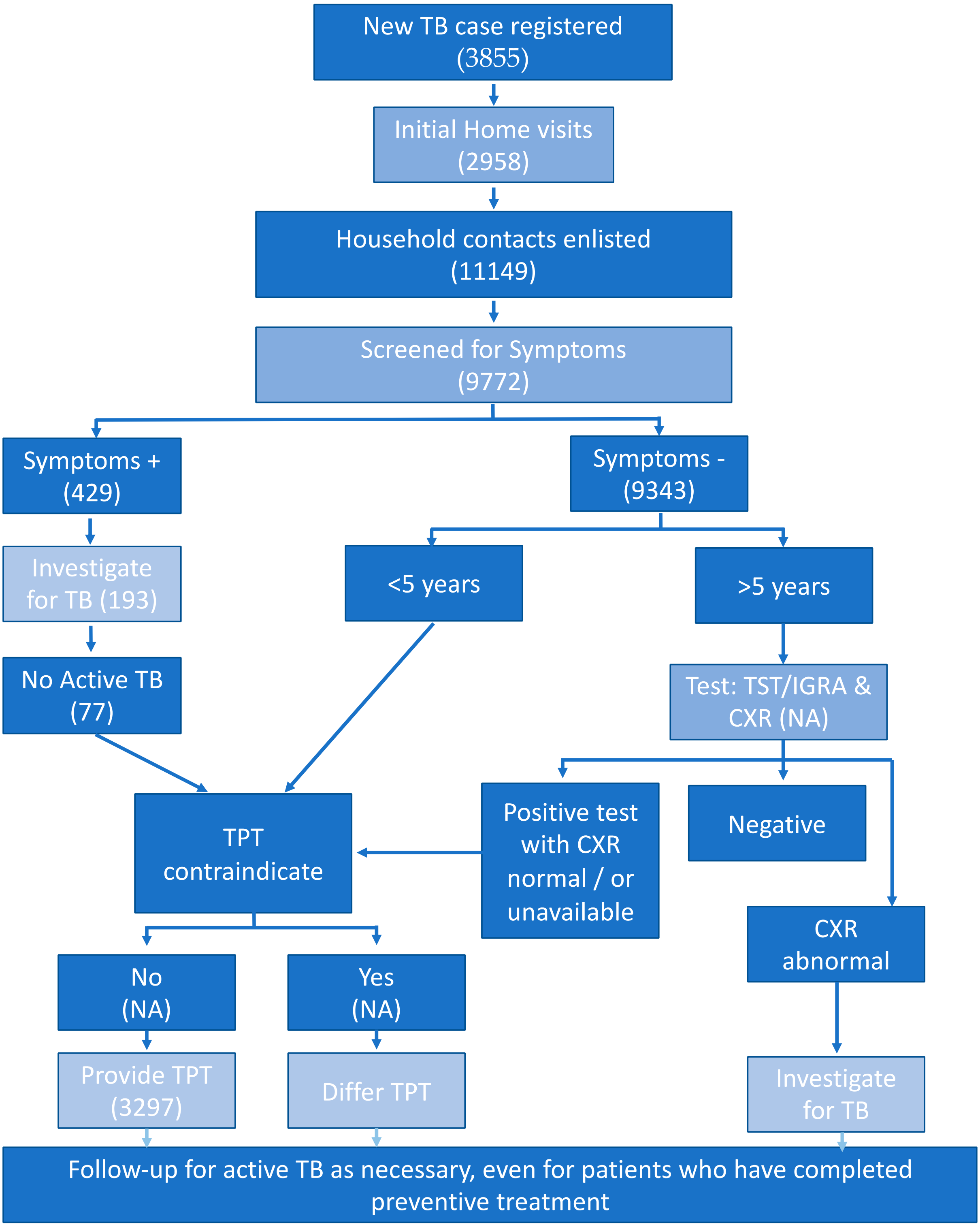

Figure S2. Flow chart showing the assessment for TPT eligibility according to guidelines for Programmatic Management of TB Preventive Treatment in India 2021.

Table S1. Variables and source of data.

**Variable**	**Source of Data**
Semi-structured checklist<br>– TPT implementation plan<br>– Mapping activity for TPT<br>– Availability of advocacy plan<br>– Staff responsible to provide TPT<br>– New staff recruited for TPT<br>– Responsibility of various staff in TPT<br>– Mechanism for Review data<br>– Community volunteers in TPT<br>– Private provider engagement<br>– Mobilization of target populations<br>– Lab test for TBI diagnosis available/ type<br>– TPT Regimen available and stock<br>– TPT adherence mechanisms<br>– ADR management plan	Interview<br><br>Observations during visit
Contacts information<br>– TPT ID<br>– Age<br>– Gender (male /female/ TG)<br>– Marital status<br>– screened for TB symptoms<br>– TST testing Positive/Negative/ Not done/ Not recorded)<br>– IGRA testing (Positive/Negative/ Not done/ Not recorded)<br>– Chest X-ray (Normal/Abnormal/Not done)<br>– TPT Eligible (yes/no)<br>– TPT started (Yes / No)<br>– TPT regimen (6H / 3HP)	Nikshay portal
Index case<br>– Nikshay ID<br>– TB category<br>– Number of members in family<br>– PHI<br>– Area<br>– Date of ATT initiation	Nikshay portal

Table S2. Operational definition of TPT cascade used in India*.

**Eligible for TPT**	All household contact of newly registered TB patient, after ruling out active TB and<br>• Child aged ≤5<br>• Child aged >5 years and adults after testing TBI positive with normal Xray or not available;<br>• PLHIV
**Started on TPT**	Eligible and without any contraindications for TPT
**Completed IPT**	Those who were started and documented to have taken six months of TPT drugs within a period of nine months from date of starting.
**Non-completion of IPT**	Those who were started and documented to have taken less than six months of TPT drugs within a period of nine months from date of starting.
**Stopped IPT**	Those who were started on TPT but was stopped due to medical (like development of adverse drug effects or development of breakthrough TB) or non-medical reasons before completion.
**Lost to Follow Up**	Participants whose outcome cannot be documented consecutively due to non-traceability for three months or more.
**Death**	Participants who died for any reason before completing IPT
**Transfer out**	Participants started on IPT but ‘transferred out’ to another DTO for any reason before completing TPT
**Breakthrough TB**	TB developed among household contact while receiving TPT, leading to a stoppage of TPT and starting of TB treatment.

* according to PMTPT guideline 2021

Annex S1. Checklist for Interview
District TB office interview

Mode of interview: Face-to-face
Briefing

Background characteristics of the respondents

Name - XXXXX Age and gender-
Designation:
Duration of the interviews – *Date of the Interview –*

Q. In your opinion, what is the status of TB in your district?
Q. After a patient is diagnosed with TB, are the household contacts screened for TB? How is the contact investigation done? What kind of challenges do you/staff face in implementation of contact investigation?
Q. How is the responses of HHC for screening, initiation and completion of TPT (Probe- average TPT completion rate and monitoring mechanism)
Q What are the common reasons for not initiating and discontinuation of TPT
Q How are the field staff overcoming/ actions taken for these issues
Q. How was your experience in implementation of TPT in your district?
Q. Can you please tell me challenges you faced?

- At administrative level
- At finance and budget
- At district level

Probe - NTEP focus on TPT implementation, its supervision and monitoring
drugs for TPT (3HP)
training of HCW (including doctors and peripheral health staff) on the latest TPT guidelines
communication and social mobilisation to generate or improve demand from the community

Q. Can you please tell me the challenges your staff faced during initial phase of implantation of TB preventive therapy?
Q Please tell me as how did you and your staff overcome those challenges?
Q. What are the programmatic issues related to initiation and continuation of TPT (related to guideline, training, logistics, manpower etc
Q. From your experience, how can we overcome those challenges and

- increase acceptance of TPT,
- reduce LFU, and
- treatment support?

Q Is there anything, you wish to say?

Debriefing –
Thankyou for the taking us to inside of the TPT program
So as per my understanding main enablers, barriers and possible solutions in the implementation of TPT are…….

Thank the participant for the valuable information

State TB officer interview

Briefing –

Background characteristics of the respondents

Name - XXXXXX   Age and gender-
Designation:

Duration of the interviews –   *Date of the Interview* –

Q. How was your experience in in implementation of TPT in your state?

Q. Can you please tell me challenges you faced?

- At administrative level
- At finance and budget
- At district and state level

Q. Can you please tell me the challenges your staff faced during initial phase of implantation of TB preventive therapy?

Q Please tell me as how did you and your staff overcome those challenges?

Q. How is the responses of HHC for screening, initiation and completion of TPT

Q What are the common reasons for not initiating and discontinuation of TPT

Q How are the field staff overcoming/ actions taken for these issues

Q. What are the programmatic issues related to initiation and continuation of TPT (related to guideline, training, logistics, manpower etc

Q. What do you think on NTEP focus on tpt implementation, its supervision and monitoring

Q. What do you think on data on TPT coverage

Q. What do you think on drugs for TPT (3HP)

Q. What do you think on collaboration between TB and other programmes on the TPT component

Q. What do you think on training of healthcare personnel (including doctors and peripheral health staff) on the latest TPT guidelines

Q. What do you think on communication and social mobilisation activities to generate or improve demand for TPT from the community

Q. From your experience, how can we overcome those challenges and

- increase acceptance of TPT,
- reduce LFU, and
- treatment support?

Q Is there anything, you wish to say?

Debriefing –

Thankyou for the taking us to inside of the TPT program
So as per my understanding main enablers, barriers and possible solutions in the implementation of TPT are…….
Thank the participant for the valuable information

Subdistrict TB manager interview

Mode of interview: Face-to-face
Briefing –

Background characteristics of the respondents
Name - XXXX Age and gender-
Designation:

Duration of the interviews – *Date of the Interview –*

Q. How was your experience in in implementation of TPT in your district?
Q. Can you please tell me challenges you faced?

- At administrative level
- At finance and budget
- At district level

Q. Can you please tell me the challenges your staff faced during initial phase of implantation of TB preventive therapy?
Q Please tell me as how did you and your staff overcome those challenges?
Q. How is the responses of HHC for screening, initiation and completion of TPT
Q What are the common reasons for not initiating and discontinuation of TPT
Q How are the field staff overcoming/ actions taken for these issues
Q. What are the programmatic issues related to initiation and continuation of TPT (related to guideline, training, logistics, manpower etc
Q. What do you think on NTEP focus on tpt implementation, its supervision and monitoring
Q. What do you think on data on TPT coverage
Q. What do you think on drugs for TPT (3HP)
Q. What do you think on collaboration between TB and other programmes on the TPT component
Q. What do you think on training of healthcare personnel (including doctors and peripheral health staff) on the latest TPT guidelines
Q. What do you think on communication and social mobilisation activities to generate or improve demand for TPT from the community
Q. From your experience, how can we overcome those challenges and

- increase acceptance of TPT,
- reduce LFU, and
- treatment support?

Q Is there anything, you wish to say?

Debriefing –
Thankyou for the taking us to inside of the TPT program
So as per my understanding main enablers, barriers and possible solutions in the implementation of TPT are…….
Thank the participant for the valuable information

Annex S2. ***Checklist Supportive supervision on TPT implementation (adopted from PMTPT 2021)***

District

**Organization structure**	When did your district/ chest clinic started TPT service?			
	How TPT is being implemented in your district?	a. PPP	b. Govt service	c. Both
	How many beneficiary needed the TPT			
	In the last quarter			
	any estimation done any time since start			
	Is MAPPING for TPT done for			
	Training of HR			
	Drugs requirement			
	Records and stationary requirement			
	Is advocacy plan available ?	a. Yes	b. No	c. don't know
	if yes then what type of activity planned	a. Mass media	b. ICE activity at HF	c. ICE activity at Community
	Who provided the drugs to beneficiaries?	a. DOTS	b. PPP	c. Both
	Is the current staff who is responsible to provide TPT is performing adequately?	a. Yes	b. No	c. don't know
	Any new staff recruited for TPT?	a. Yes	b. No	
	If not anyone in pipeline?			
	Who is given responsibility for			
	•Screening of all target population			
	•Counsel and refer those with symptoms of TB for TB diagnosis to TB detection centres			
	•assessment for TBI eligibility			
	to provide treatment			
	• counselling (at home / tele/video calls) For adherence, signs/symptoms of TB, adverse drug reactions			
	•Update data on Prevent TB India app/Nikshay TPT module			
	Training of staff			
	STS is responsible for			
	Mechanism for Review data			
	Nikshay portal	a. Yes	b. No	c. don't know
	If yes frequency			
	Prevent TB India app	a. Yes	b. No	c. don't know
	If yes frequency			
	TPT register	a. Yes	b. No	c. don't know
	If yes frequency			
	Any other			
	When was last done		Days ago	
	provide feedback to PHC/ UPHC			
**Training**	Have you attended any training on PMTPT	a. Yes	b. No	c. don't remember

	Any training in last 6 month		a. Yes	b. No		
	Are you aware of Programmatic Management Guideline For TB Preventive Therapy, 2021		a. Yes	b. No	c. Somewhat	
	Are you satisfied with the training		a. Yes	b. No	c. Somewhat	
	Who are the TPT target populations					
**Community engagement plan and demand generation activities**	Are Community volunteers (TB survivors/ champions, ASHAs & AWWs)					
	aware about TPT					
	trained about TPT					
	Mobilization of target populations (contacts/ vulnerable/high-risk groups) for TPT even held		a. Yes	b. No	c. don't know	
	Reasons :					
	Suggested solutions:					

**Diagnostics**	If Lab test for TBI diagnosis		a. Present	b. Not present	c. Don't know		
	If yes, TBI test offered		a. IGRA	b. TPT	c. Chest X-ray		
	If no, whether provision of outsourcing of TBI present		a. Yes	b. No			
	Which test		a. IGRA	b. TPT	c. Chest X-ray		
**Drugs and log management**:	TPT drugs available		a. Yes	b. No			
	Regimen		a. 6H	b. 3HE	c. Others…		
	Drugs stocks supplies for (for line listed pts)	6H	a. 1 year	b. 6 months	c. 3 months	d. 1 months	e. No stock currently
		3HE	a. 1 year	b. 6 months	c. 3 months	d. 1 months	e. No stock currently
	Lab Supplies available for (for estimated pts)		a. 1 year	b. 6 months	c. 3 months	d. 1 months	e. No stock currently
	Is space identified for drugs storage		a. Yes	b. No	c. don't know		
	Upgradation of storage space to store TPT drug quantities done		a. Yes	b. Already adequate	c. to be done		
	4.8 TPT adherence mechanisms		a. Regular home visit/	b. Regular telephonic con /	c. Pill counts during collection of new strips/	d. No provision	
	If yes, frequency of adherence assessment		a. Weekly	b. Fortnightly	c. Monthly	d. Once only	
	ADR management plan present		a. Yes	b. No	c. don't know		
	4.9 Supplies for recording & reporting (printing, digital		a. Yes	b. No	c. don't know		

	platform readiness with peripheral devices and internet) available	
**Private provider engagement**:	Level of Private provider engagement:	a. For test and treat; b. only treat; c. only test; d. community mobilization; e. None
	Type of Private provider engagement:	1. Patient Provider Support Agency (PPSA)
		2. Public Health Action (Counselling and adherence management, Contact tracing and chemoprophylaxis)
		3. Specimen Management
		4. Diagnostics
		5. Treatment Services
		6. Drug Access and Delivery Services
		7. Active TB Case Finding and TB Prevention
		8. Advocacy, Communication and Community Empowerment

Checklist of TPT implementation at State Level

1	**State TPT committee**	Established	Not established		
2	**Mapping activity done**	Yes	No	Planned	
3	**TPT implementation plan in annual PIP**	Present	No separate plan		
4	**Budget for TPT allocated**	Present	No separate budget		
5	**Line list available**	Yes	No		
6	**Mechanism for TBI screening**	Establishment	Not establishment		
7	**TPT is being implemented**	Public	Private	Both	
8	**Advocacy plan available**	Yes	No	working on it	
9	**IEC activities planned**	Mass media	Health facility	community	None
10	**Active case finding**	Being conducted	Not done	Planned	
11	**PPP engagement**	only treat	only test	For test and treat	community mobilization
12	**Human resources Adequate**	Adequate for TPT service	Not adequate for TPT	Not fulfilling to NTEP norms	
13	**Deficiency at**	a. Screening	b. Counsel and refer	c. provide treatment	
		d. Monitor & adherence assessment	e. Data management	f. supportive supervision	
14	**HR trained**	All trained	Majority trained	Not trained	
15	**Mechanism for Review data**	Nikshay portal	Prevent TB India app	TPT register	None
16	**Community volunteers aware about TPT**	Yes	No		
17	**TBI test available**	Yes	No		
18	**IGRA**	Available	Not available		
19	**TPT**	Available	Not available		
20	**Provision of outsourcing of TBI diagnostic**	Yes	No		
21	**Regimen**	6H	3HE	Others	

22	**Drugs stocks Supplies**	Adequate	Not adequate		
23	**Lab Supplies**	Adequate	Not adequate		
24	**TPT adherence mechanisms**	Home visit	telephonic	Pill counts	No provision till now, working on it
25	**ADR management**	In place	Not available		
26	**Supplies for recording & reporting**	Adequate	Not adequate		
